# Magnetic resonance imaging of parotid gland tumors: a pictorial essay

**DOI:** 10.1186/s12880-022-00924-0

**Published:** 2022-11-07

**Authors:** Soung Yung Kim, Urs Borner, Jung-Hyun Lee, Franca Wagner, Dechen W. Tshering Vogel

**Affiliations:** 1 Röntgeninstitut Marktgasse AG, RIMED AG, Marktgasse 6/8, 3011 Bern, Switzerland; 2 Department of Otorhinolaryngology - Head and Neck Surgery, Inselspital, Bern University Hospital, University of Bern, 3010 Bern, Switzerland; 3 Department of Life Science, University of Seoul, Seoul, Republic of Korea; 4 Department of Diagnostic and Interventional Neuroradiology, Inselspital, University Hospital Bern, University of Bern, Freiburgstrasse 10, 3010 Bern, Switzerland; 5 University Institute for Diagnostic, Interventional and Pediatric Radiology, Inselspital, University Hospital Bern, University of Bern, Freiburgstrasse 10, 3010 Bern, Switzerland

**Keywords:** Magnetic resonance imaging, Benign parotid gland tumors, Malignant parotid gland tumors, Imaging features

## Abstract

Imaging of parotid gland tumors is challenging due to the wide variety of differential diagnoses. Malignant parotid tumors can have very similar features to benign ones, such as slow growth and displacement instead of infiltration of neighboring structures. Malignant and benign tumors may therefore not be clinically distinguishable. Correct characterization of parotid tumors (i.e., benign or malignant) determines preoperative treatment planning and is important in optimizing the individualized surgical plan. Magnetic resonance imaging (MRI) is the imaging modality of choice for evaluation of suspected parotid gland lesions and differentiation between benign and malignant lesions. Certain conventional MRI features can suggest whether a mass is more likely to be a benign or low-grade malignancy or a high-grade malignancy and adding diffusion-weighted imaging or advanced MRI techniques like perfusion can aid in this distinction. Morphological features seen on MRI, such as low signal on T2-w, infiltrative changes or ill-defined margins, change over time and diffusion restriction can point to the malignant nature of the lesion. MRI is useful for detection and localization of the lesion(s), and associated findings like perineural spread of tumor, lymph node involvement and infiltrative changes of the surrounding tissues. In this pictorial essay, we present selected images of a variety of benign and malignant parotid tumors and emphasize the MRI features that may be useful in their characterization.

## Background

Salivary gland tumors are rare, accounting for roughly 2–6.5% of all head and neck tumors and about 0.5% of all malignancies [1–3]. The most common location of salivary gland tumors is the parotid gland (70%), followed in descending order by the submandibular gland, the minor salivary glands, and the sublingual gland [3].

Parotid gland tumors are complex with a wide variety of histological types of benign and malignant lesions. Most of the parotid tumors in adults (about 80%) are benign, the most frequent being pleomorphic adenomas (about 65%) followed by Warthin’s tumors (about 15–20% of all parotid gland tumors) [4]. The most common malignant tumors are mucoepidermoid carcinomas, which account for 10% of salivary gland tumors and 30% of malignancies [4].

Preoperative characterization of parotid gland tumors using dedicated imaging is crucial for appropriate treatment planning, as the surgical approach is different for benign and malignant lesions and depends also on the localization of the tumor. Extracapsular dissection or partial parotidectomy is the surgical procedure of choice in patients with benign lesions. Patients with malignant tumors need more aggressive surgery (i.e., lateral, subtotal or total parotidectomy) with or without a neck dissection and potential sacrifice of the facial nerve [5]. Clinical assessments have limitations in diagnosing malignant parotid neoplasms and in most cases of palpable parotid masses, differentiation between benign and malignant lesions is not possible by clinical examination only. In addition, fine needle aspiration biopsy is sometimes inconclusive and may provide insufficient results.

Although ultrasound is also very useful for evaluation of parotid lesions, the deep lobe is not adequately seen using this technique. Therefore, magnetic resonance imaging (MRI) has assumed a major role in assessing lesions of the parotid gland. MRI can also provide more accurate information about the localization of the tumor either in the superficial or deep lobe. Although computed tomography (CT) can also provide this information, the better soft tissue resolution of MRI allows more accurate delineation of margins as well as tissue characterization. In this pictorial essay, we present images of a variety of benign and malignant parotid gland tumors and emphasize the MRI features that may be useful in their characterization and differentiation. The overview of the various figures is presented in Table 1.

**Table 1:** Overview of the images

Figure no	Benign lesions	Figure no	Malignant tumors
1	Pleomorphic adenoma	7	Mucoepidermoid carcinoma
2	Warthin’s tumor	8	Adenoid cystic carcinoma
3	Oncocytoma	9	Acinic cell carcinoma
4	Infantile hemangioma	10	Parotid carcinoma
5	Lipoma	11	Poorly differentiated carcinoma
6	Benign lymphoepithelial cyst	12	Lymphoma

## Benign tumors

### Pleomorphic adenomas

Pleomorphic adenoma (benign mixed tumor) is the most common benign parotid gland tumor in adults and predominates in females (2:1); it usually presents as a slowly growing, asymptomatic mass in a middle-aged patient. Pleomorphic adenoma has a tendency to degenerate, which is dependent on the age of the tumor [6].

**Fig. 1:**
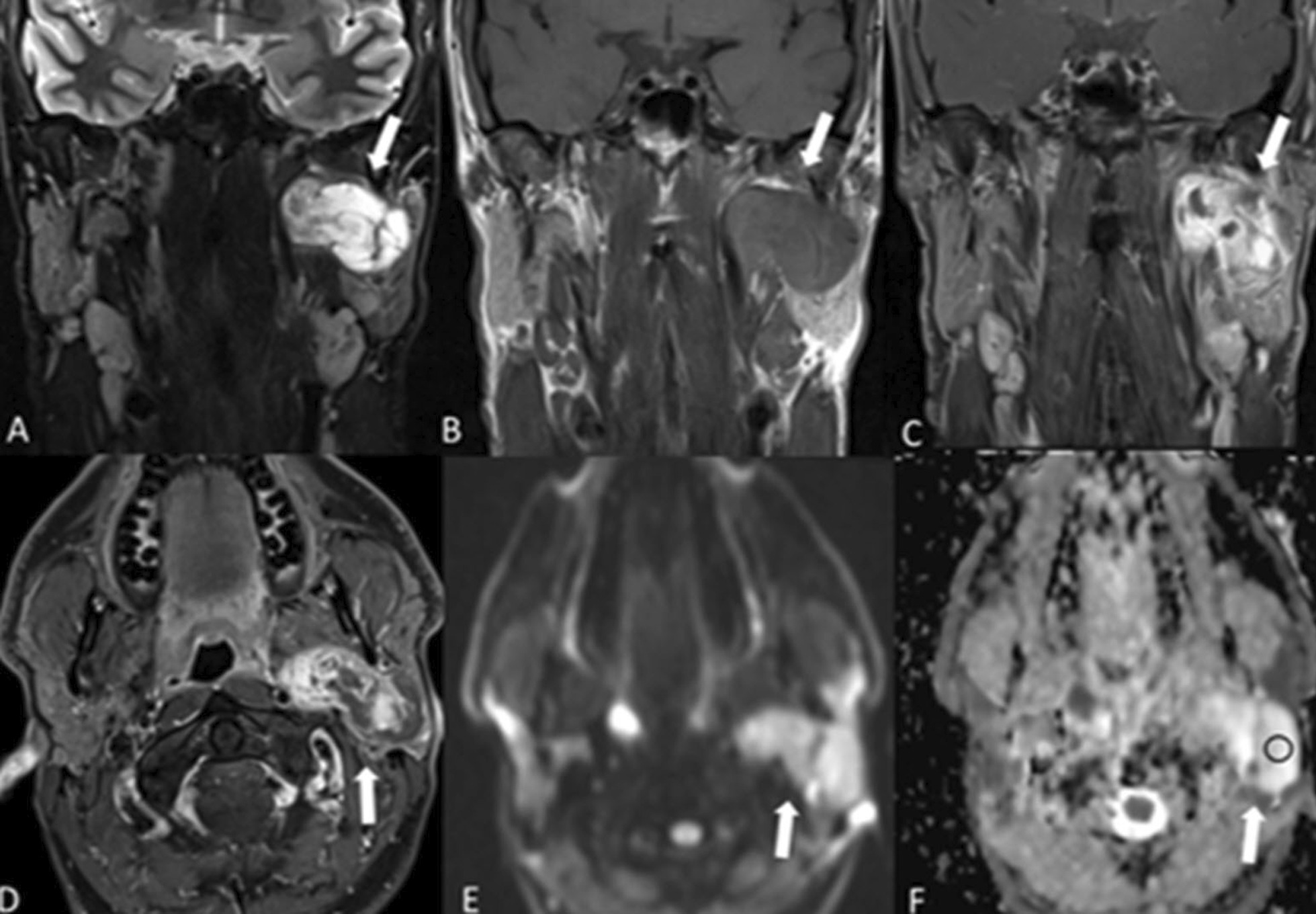
Pleomorphic adenoma. A 39-year-old male presenting with a painless preauricular swelling on the left side, which was proven to be a pleomorphic adenoma. Coronal T2-w MRI (**A**) shows a predominantly hyperintense lesion of the left parotid gland (arrow), which is hypointense on T1-weighted image (**B**). The lesion lies in both the deep and the superficial parotid with an hourglass configuration. The coronal (**C**) and axial (**D**) images after injection of intravenous gadolinium with fat saturation shows heterogeneous enhancement of the lesion. The signal intensity is high in the diffusion-weighted image (**E**) and it remains high in the ADC maps (**F**) with ADC values (o) of 2300 ± 270 × 10^−6^ mm^2^/s and this is helpful for identifying pleomorphic adenoma

The alternative term, benign mixed tumor, reflects the histological heterogeneity suggesting a variety of imaging findings [5]. The imaging appearances vary depending on tumor size. Small tumors are more homogeneous and well defined, with strong enhancement following administration of intravenous contrast medium. Larger tumors tend to have pedunculated outgrowth from the main lesion (lobulated contour) and are more heterogeneous, with necrotic and hemorrhagic areas [5]. In general, however, the pleomorphic adenoma has very high signal on T2-weighted (T2-w) images, higher than the cerebrospinal fluid signal (Fig. 1) with higher apparent diffusion coefficient (ADC) values. Dystrophic calcification is highly suggestive of this tumor but cannot be visualized or distinguished from focal areas of fibrosis on MRI [7].

There are three malignancies associated with pleomorphic adenoma: (1) the carcinoma ex pleomorphic adenoma; (2) the true malignant mixed tumor (carcinosarcoma); and (3) the metastasizing ´benign’ mixed tumor. The carcinoma ex pleomorphic adenoma is a malignant change of a benign mixed tumor or a malignant tumor in a patient who has previously undergone surgical tumor resection for a pleomorphic adenoma. The true malignant mixed tumor (carcinosarcoma) is a rare entity with a poor prognosis. The metastasizing ‘benign’ mixed tumor is the rarest variant. Metastases may be multiple and occur in the lung, bone and soft tissue, often over decades [5].

### Warthin’s tumor

Warthin’s tumor, also known as cystadenolymphoma, is the second most common benign tumor of the parotid gland. It is the most common neoplasm that manifests as multiple lesions and is bilateral in up to 10–20% of cases [5, 8]. It often presents as a slowly growing painless mass in older men and has a strong association with smoking [9].

**Fig. 2:**
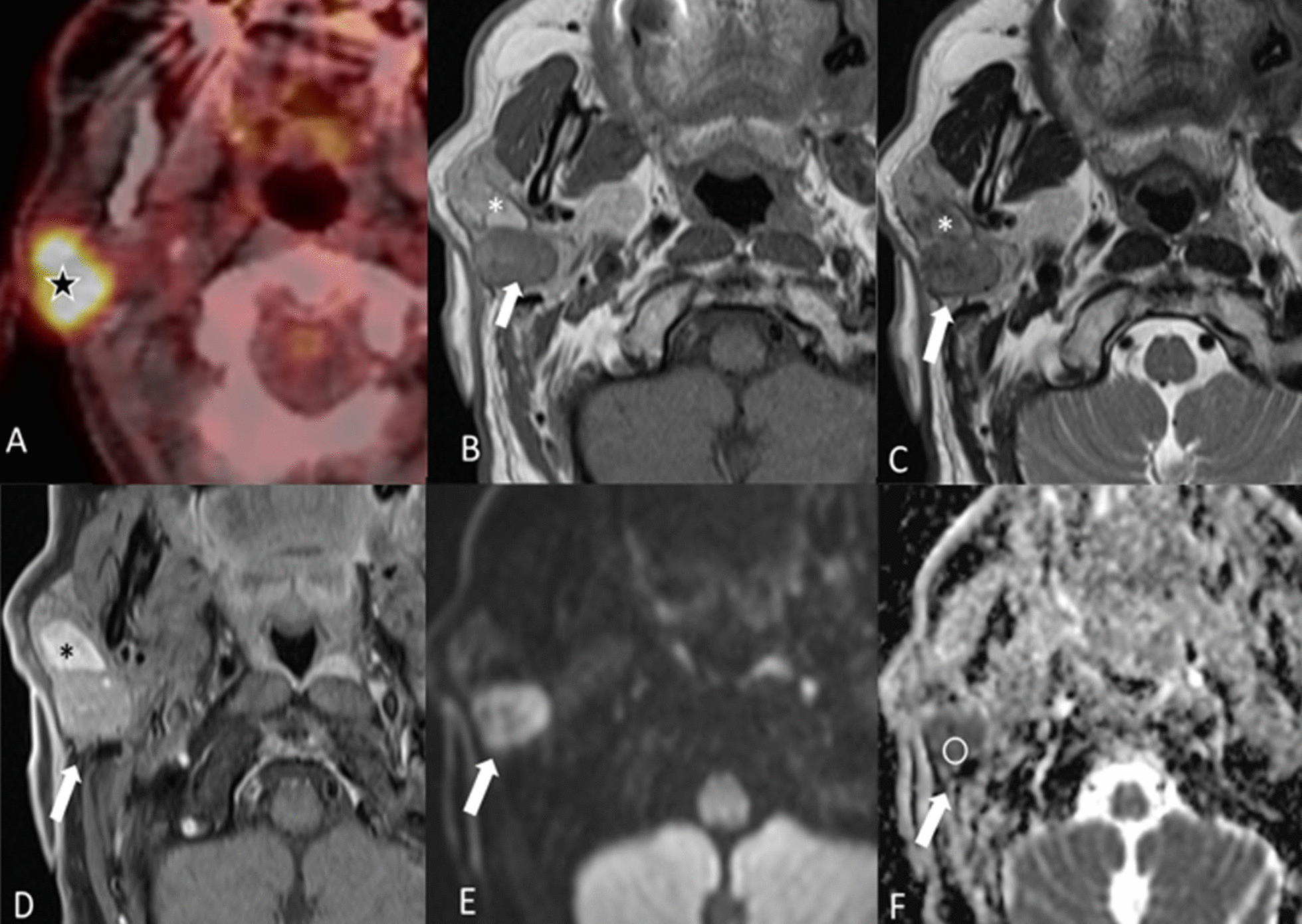
Warthin’s tumor. A 73-year-old male with malignant melanoma of the back who underwent an ^18^F-FDG PET/CT scan for staging. The PET/CT scan (**A**) showed a highly metabolic lesion in the right parotid gland (star). Fine needle aspiration cytology revealed a Warthin’s tumor. The dorsal part of the lesion is predominantly hypointense on both T1-w axial MRI (**B**) and T2-w axial MRI (**C**) with slightly heterogeneous enhancement after injection of contrast as seen on the post-contrast fat saturated T1-w image (**D**). There is diffusion restriction of the enhancing part with a hyperintense signal on the high b value diffusion-weighted image (**E**) and a low signal on the ADC map (**F**). The ventral portion of the lesion (white asterisk) is hypointense on T2-w and hyperintense on T1-w due to some bleeding caused by the fine needle aspiration cytology, which had been performed prior to the MRI

Warthin’s tumors are seen in imaging exams as well-encapsulated, partly cystic, partly solid lesions, often located in the parotid tail. On MRI, Warthin’s tumors show a low to intermediate T1-w signal intensity with cysts containing cholesterol components appearing as focal areas of high signal intensity [10]. On T2-w images, Warthin’s tumors are heterogeneous with variable signal intensity. After contrast medium administration, the solid components of the tumor can enhance minimally (Fig. 2). Warthin’s tumors are also often picked up incidentally during ^18^F-fluorodeoxyglucose positron emission tomography CT (^18^F-FDG PET/CT) scans as they take up glucose [11] (Fig. 2).

### Oncocytoma

Parotid gland oncocytoma is a rare benign epithelial tumor that accounts for less than 1% of all salivary gland tumors [8, 12–14]. The most common clinical presentation is a painless, slow-growing, non-tender, lobulated, and mobile mass. Parotid gland oncocytomas can occasionally (< 10%) be multifocal or bilateral [13].

**Fig. 3:**
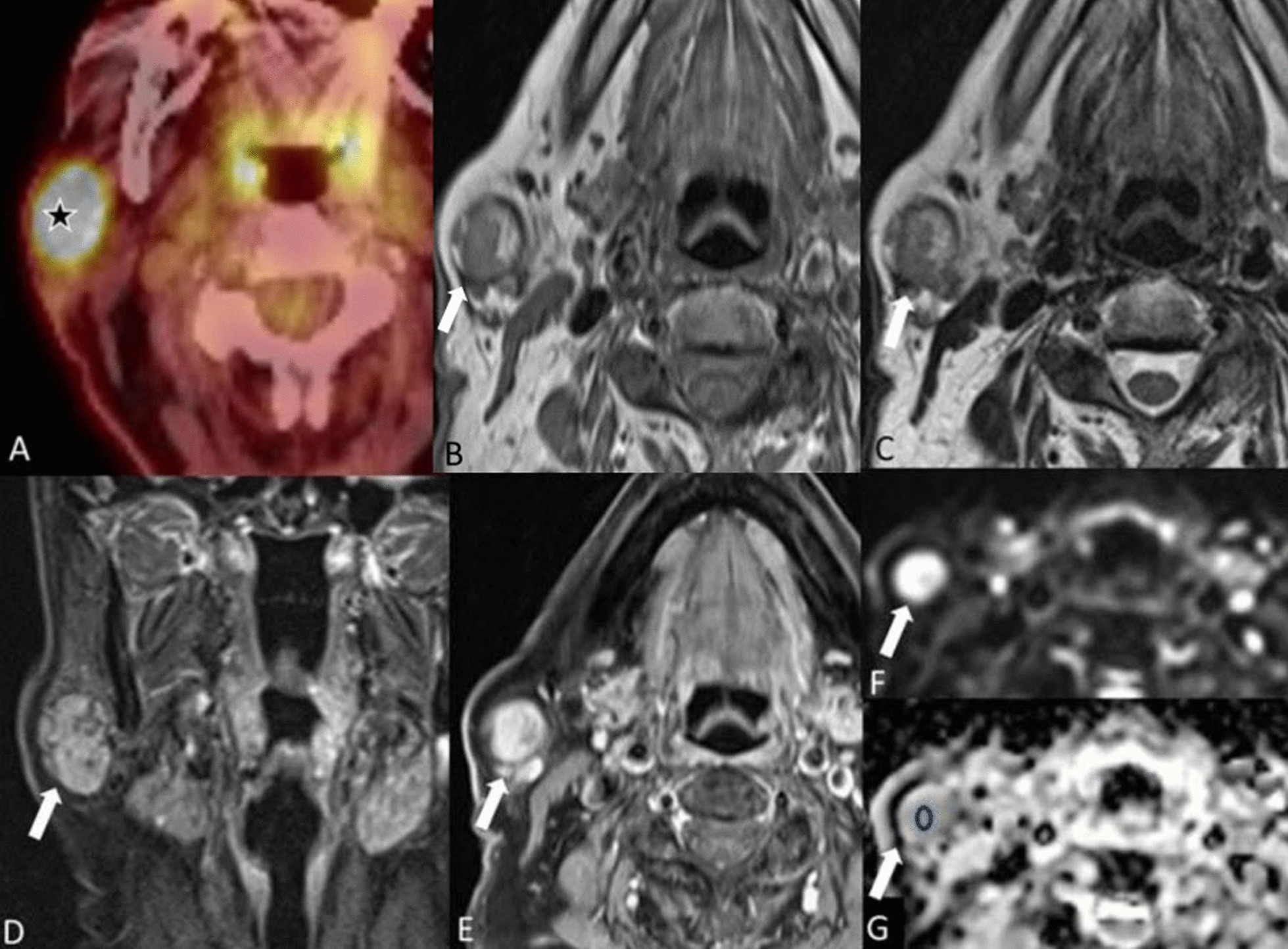
Oncocytoma. An 83-year-old female with melanoma of the right arm. ^18^F-FDG PET/CT (**A**) revealed a strongly glucose avid lesion in the right parotid gland. The lesion shows heterogeneous signal intensity on both T1- (**B**) and T2-w (**C**) images. The coronal turbo inversion recovery magnitude (TIRM) image (**D**) shows the slightly lobulated structure. There is heterogeneous enhancement of the lesion on the T1-w fat saturated image after gadolinium injection (**E**) and slight diffusion restriction as seen on the b800 image (**F**) with high signal intensity and corresponding low signal intensity (o) in the ADC map (**G**)

On MRI examination, oncocytomas are generally hypointense on T1-w images, isointense to hypointense on T2-w images and isointense on T1-w fat-saturated images (Fig. 3). They are also metabolically active and can show avid uptake of glucose on PET scans [14]. Oncocytomas tend to have higher ADC values due to lower diffusion restriction than in Warthin’s tumor and this can help in differentiating between these two tumors.

### Infantile hemangioma

Parotid infantile hemangiomas are the most common benign parotid tumor in children with a predominance in females of 3:1 [15]. Hemangiomas manifest shortly after birth as unilateral parotid swelling. They show rapid enlargement in infancy, which is normally followed by gradual regression starting at 8–18 months of age [14]. They can occasionally act as significant vascular shunts if they do not involute.

**Fig. 4:**
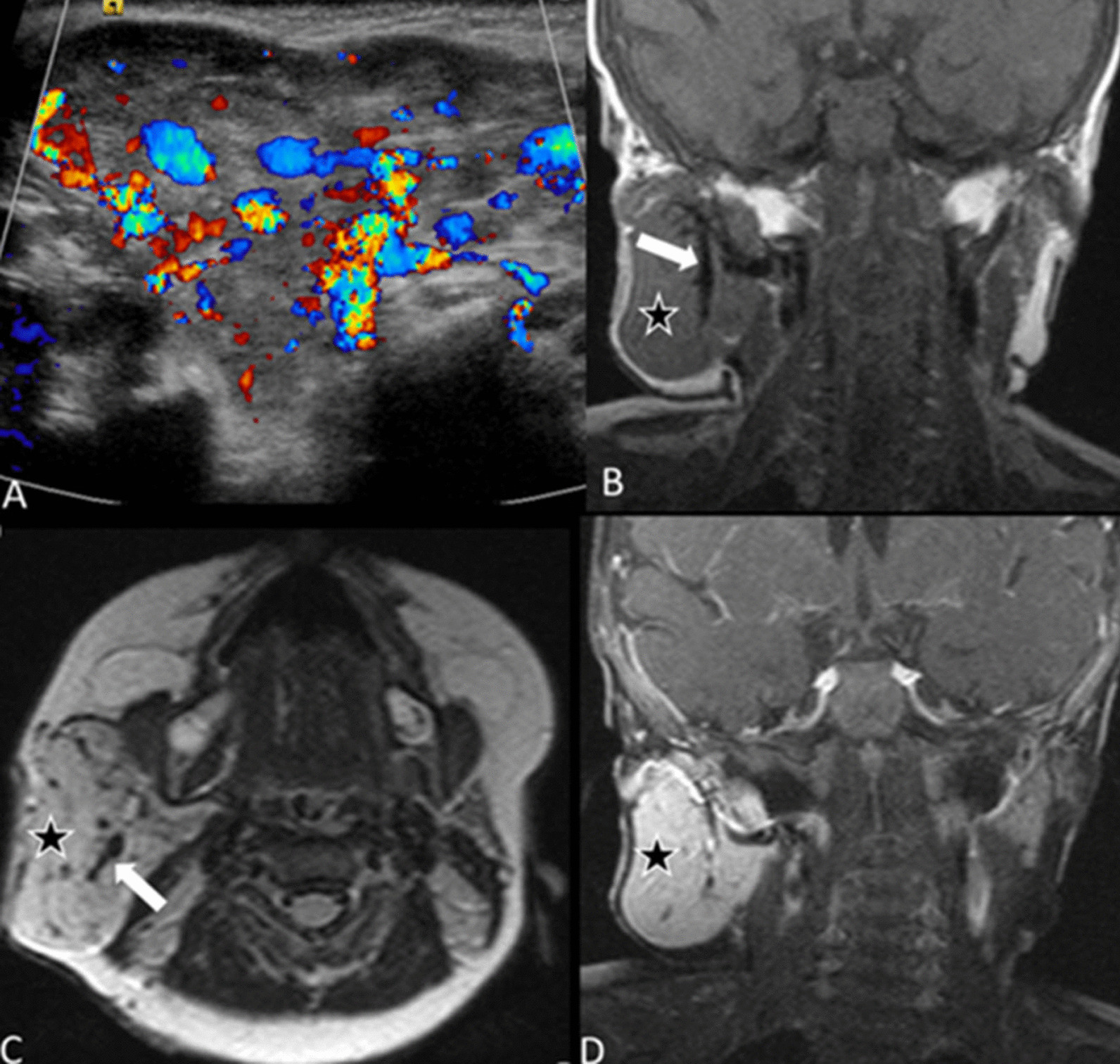
Infantile hemangioma. A 4-month-old female child with a hemangioma (star) on the right side, present since birth, with a marked increase in the size over the previous 2 months. Doppler ultrasound (**A**) shows the characteristic vascular nature of the mass. The lesion is hypointense on T1-w (**B**), hyperintense on T2-w (**C**) with diffuse homogeneous enhancement (**D**). Note the prominent vessels with flow voids in the lesion (white arrows)

On MRI, parotid infantile hemangiomas are isointense to muscle on T1-w images and hyperintense on T2-w images. After contrast medium administration, there is homogeneous contrast enhancement with the presence of characteristic flow voids in or around the mass, due to prominent vasculature [5] (Fig. 4).

### Lipoma

**Fig. 5:**
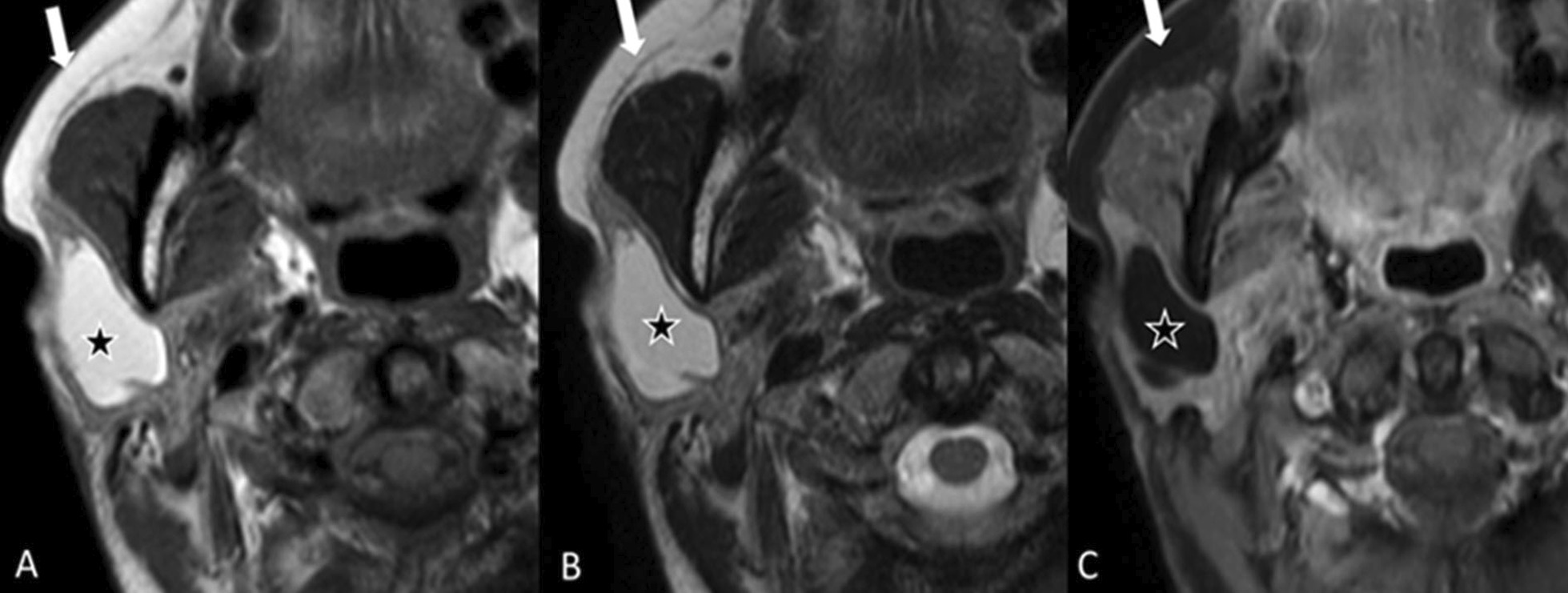
Lipoma. An 82-year-old female with an incidental finding of lipoma. Axial T1‑w (**A**) and axial T2‑w (**B**) MRI demonstrates a well‑circumscribed high signal lesion in the superficial lobe of the right parotid gland (white arrows) similar to that of subcutaneous fat. Axial T1‑weighted post-contrast fat‑suppressed MRI (**C**) shows lack of enhancement with a typical hypointense signal isointense to fat (white arrow)

Parotid lipomas are uncommon, well-circumscribed, benign non-epithelial neoplasms. They may be located with the gland itself or may be extracapsular within the adjacent parotid space [16]. They show the characteristic signal of fat-containing lesions on MRI [17]. Typical signal intensities isointense to fat on all pulse sequences are visible on MRI (Fig. 5). It is important to differentiate lipomas from other lipomatous lesions of the parotid gland, such as liposarcoma.

### Tumor-like lesions: benign lymphoepithelial lesions

Benign lymphoepithelial lesions are caused by reactive lymphoid infiltrate with follicular hyperplasia, which leads to proliferation and disruption of ductal epithelium and obliteration of the acinar glandular tissue [18]. They are normally seen in people with Sjögren’s syndrome in the context of lymphoepithelial sialadenitis or HIV infection [19]. On imaging, numerous entirely cystic, combined cystic and solid, or entirely solid lesions, which can be either bilateral or unilateral, are observed in the parotid gland [20].

Sporadic or simple lymphoepithelial cysts (LECs) of the parotid gland are not associated with HIV or Sjögren’s syndrome. They are predominantly unilocular, unilateral, solitary and well circumscribed [20, 21].

**Fig. 6:**
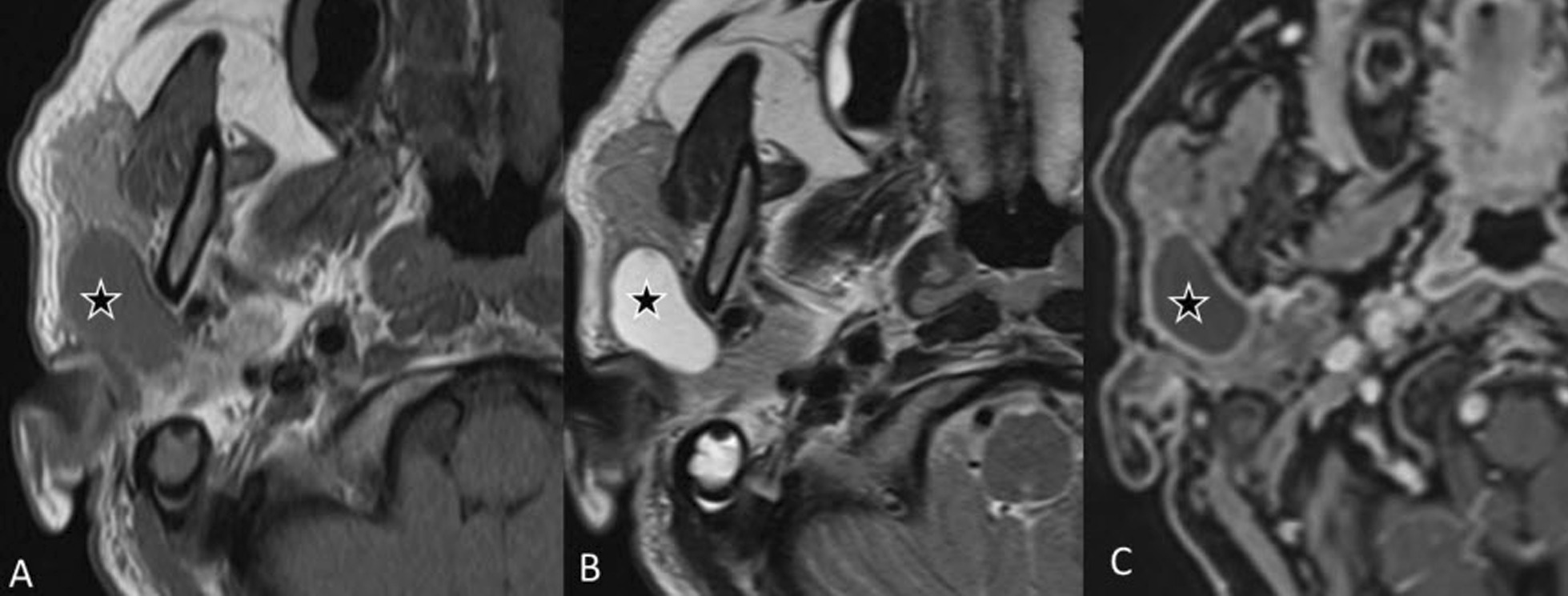
Sporadic/simple lymphoepithelial cyst. A 63-year-old female with painless swelling in the right cheek. Axial T1‑w (**A**) and axial T2‑w (**B**) MRI demonstrate a well‑circumscribed unilocular cystic lesion in the superficial lobe of the right parotid gland (star). Axial T1‑w postcontrast fat‑suppressed MRI (**C**) shows a smooth, thin rim enhancement of the lesion

On MRI, LECs of the parotid gland are usually well-circumscribed cystic spaces and may demonstrate thin rim enhancement following contrast administration (Fig. 6). Other cystic lesions in the parotid gland, such as first branchial cleft cysts (FBCC), salivary duct cysts and sialoceles can look similar. Differentiation on MRI is possible if additional features can be identified.

FBCCs in the parotid gland can present as a cystic mass adjacent to the external auditory canal. They are usually well defined, unilocular or multilocular, oval or round and thin-walled in the absence of complications like infections or bleeding. They can be difficult to differentiate from LECs: the presence of fistulas and sinuses points to a diagnosis of FBCC [20].

Salivary duct cysts can be seen in the superficial lobe of the parotid gland. These are considered to be true cysts and can be congenital or acquired. They are painless, unilateral swellings which are compressible. They appear similar to the LECs but may occasionally have fluid-fluid levels and mural nodules [20].

A sialocele has been described as an accumulation of extravasated saliva in the surrounding tissue secondary to excretory duct obstruction following trauma or surgery. MRI shows well-defined cystic lesions, but the signal intensity of the fluid can vary according to the mucin content of the sialocele [20].

## Malignant tumors

**Fig. 7:**
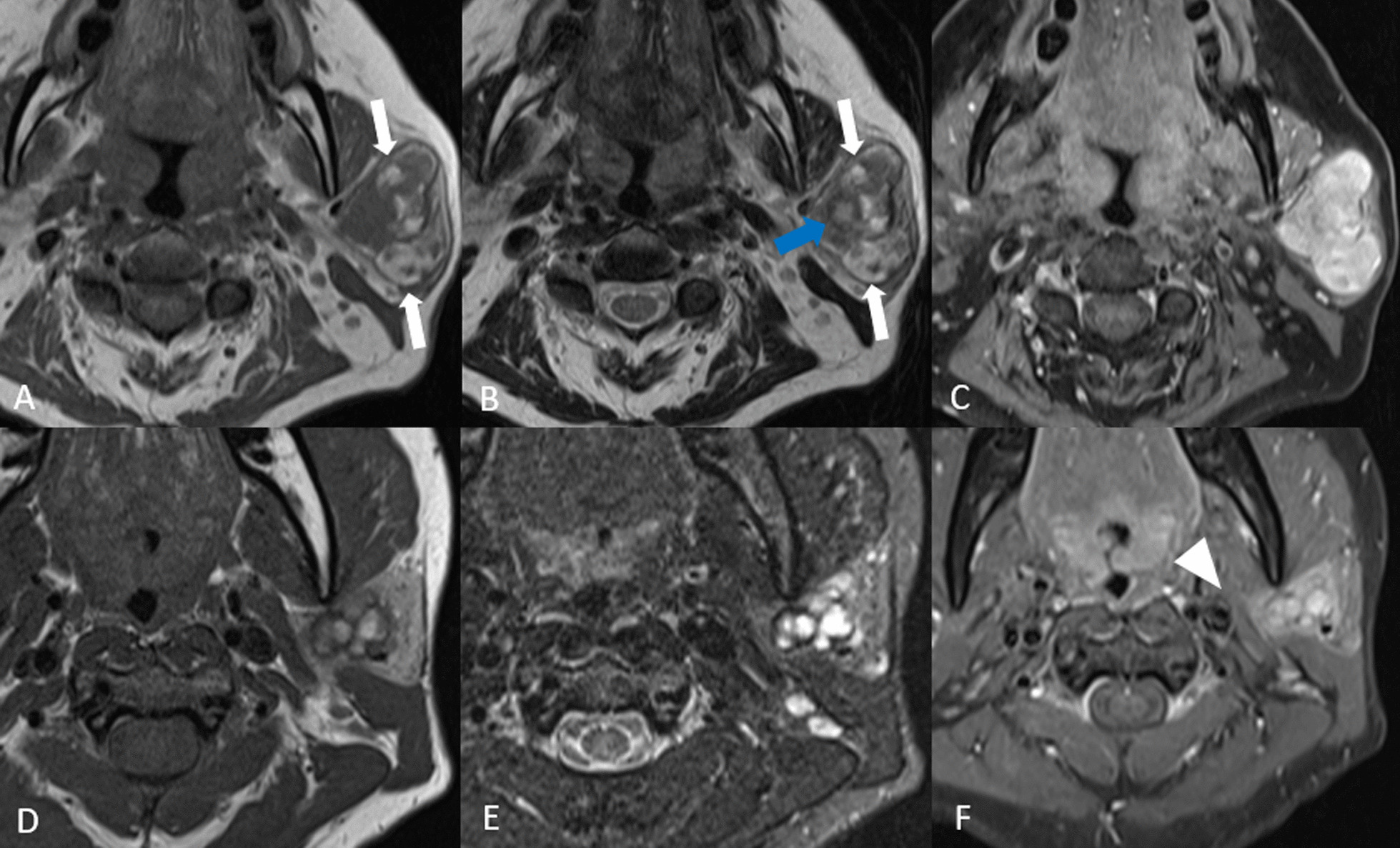
Mucoepidermoid carcinoma, two cases. Patient 1 (**A**–**C**): An elderly patient had a low-grade mucoepidermoid carcinoma in a pleomorphic adenoma. The T1-w image without fat saturation (**A**) shows a heterogeneous lobulated lesion in the left parotid gland with rounded high signal areas on the native scans (white arrows). The T2-w image (**B**) also shows a similar area reflecting high protein content (white arrows) and additional cystic areas (blue arrow) with the usual fluid intensities. The T1-w fat saturated image (**C**), after administration of intravenous contrast, shows enhancement of the solid portions. There was little diffusion restriction (no images added). Patient 2 (**D**–**F**): A 31-year-old woman with a mucoepidermoid carcinoma of the left parotid gland. The axial T1‑w image (**D**) and axial TIRM image (**E**) show a heterogeneous lesion with multiple small cysts involving both the deep and the superficial lobe of the left parotid gland. The T1-w contrast medium enhanced fat suppressed image after administration of contrast (**F**) shows strong enhancement and slightly irregular infiltrating margins in the anteromedial part of the mass (white arrowhead)

**Fig. 8:**
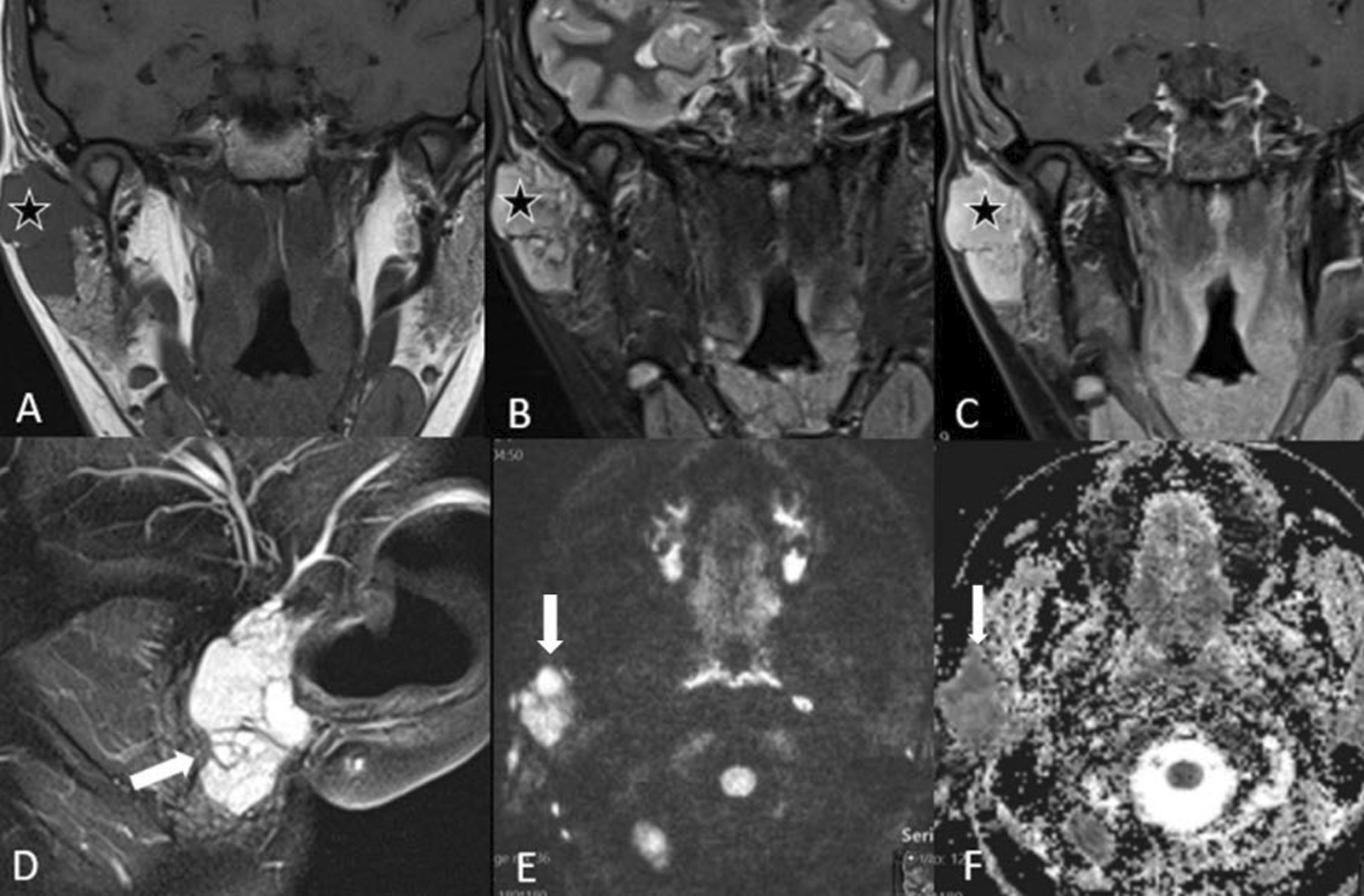
Adenoid cystic carcinoma. A 38-year-old female presented with a painful preauricular swelling on the right side. Coronal T1-w MRI (**A**) shows a homogeneously hypointense mass (star) in the superficial lobe of the right parotid gland, which is hyperintense on the coronal T2-STIR MRI (**B**) and shows strong and homogeneous enhancement on the post-contrast T1-w image with fat suppression (**C**). The lesion is lobulated (arrow) as seen in the sagittal T2 image through the parotid gland (**D**). The lesion shows high signal intensity (arrow) on the high b value image (**E**) with a corresponding low signal (arrow) on the ADC map (**F**) indicating diffusion restriction. The parotid gland on the left side does not show any increase in signal intensity in the high b value image (**E**)

**Fig. 9:**
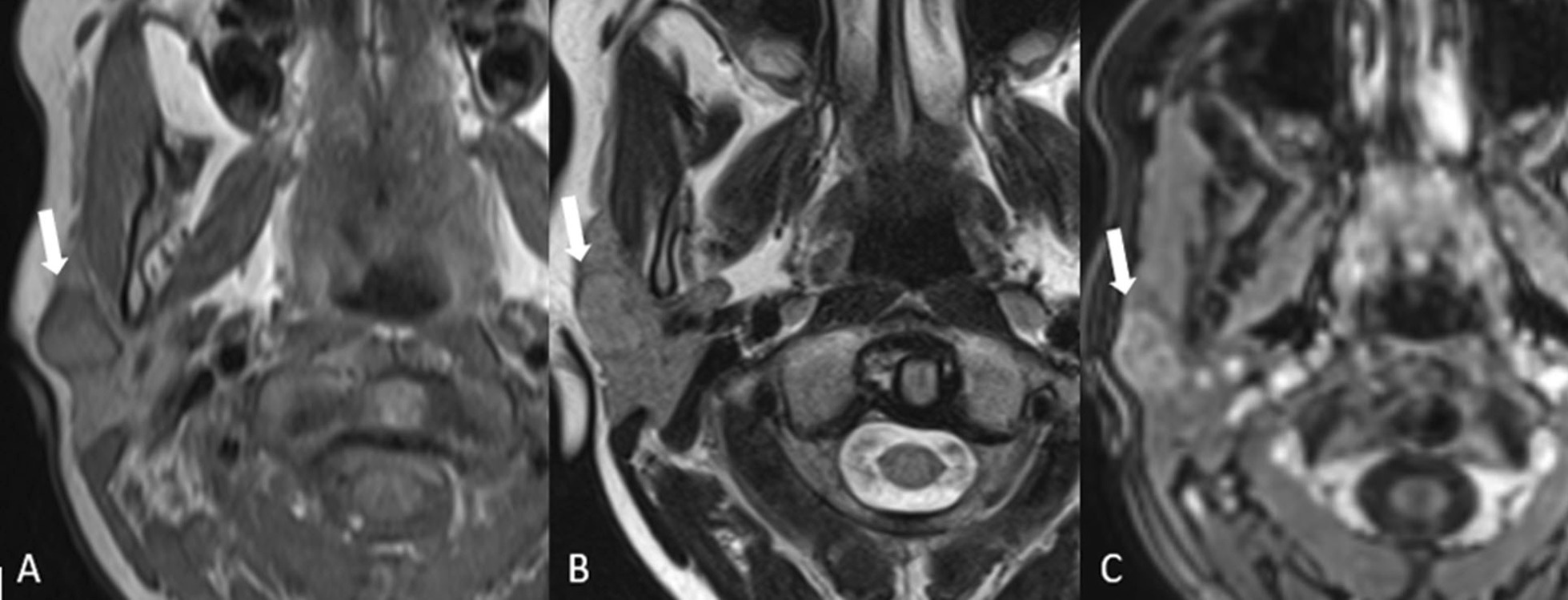
Acinic cell carcinoma. A 10-year-old female with a painless and slowly growing palpable parotid mass on the right side (white arrows) with the clinical suspicion of a lateral neck cyst. Axial T1-w MRI (**A**) shows a well-defined lesion in the right parotid gland relatively hypointense to the parotid tissue. The lesion is only slightly brighter than the parotid tissue in the axial T2-w (**B**) MR image. There is some heterogeneous enhancement on the T1-w fat sat MRI after administration of contrast (**C**)

**Fig. 10:**
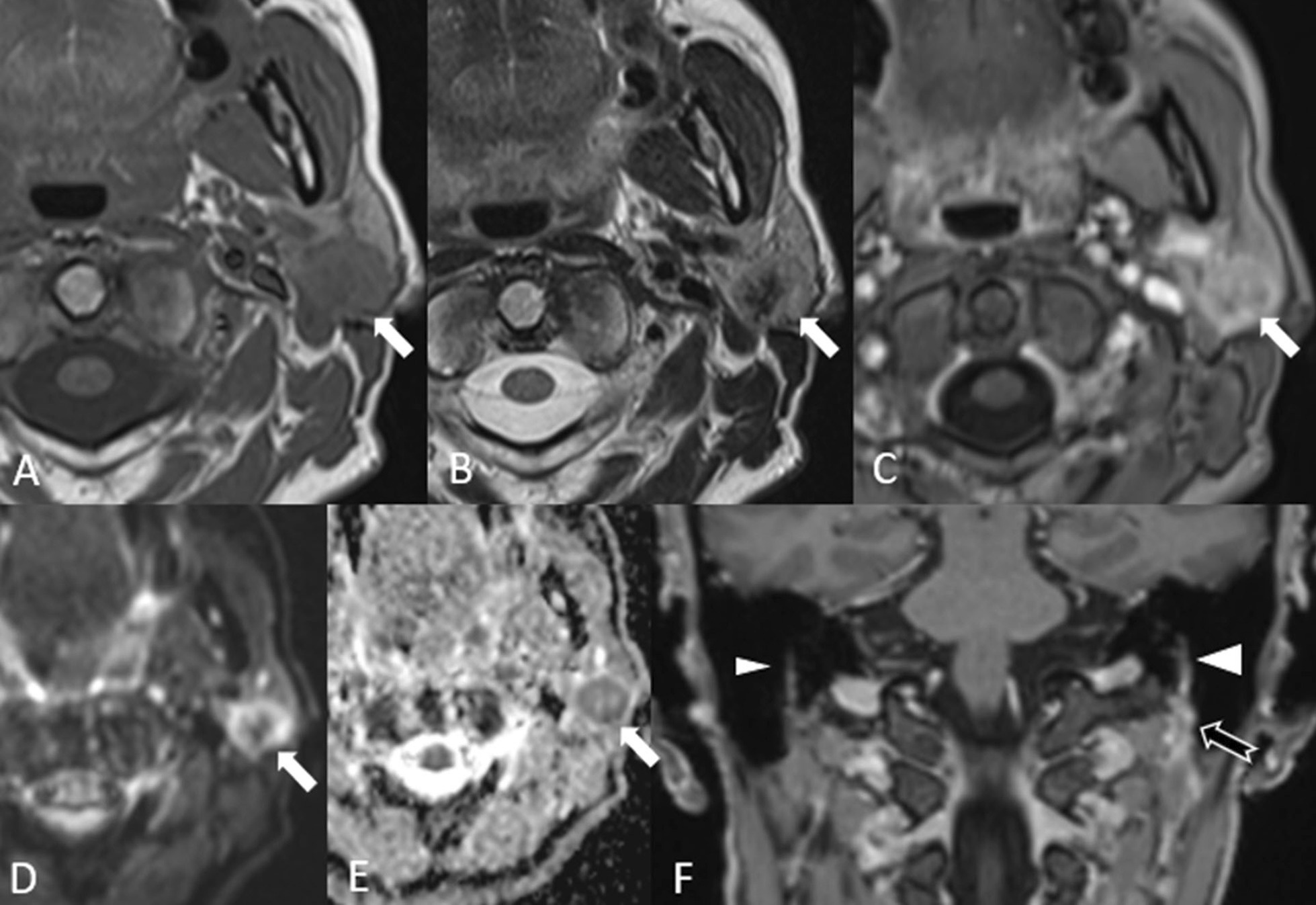
Parotid carcinoma. A 63-year-old female with facial paralysis on the left side. There is a lobulated hypointense lesion in the left parotid gland (arrow) on the T1-w axial image (**A**). The lesion shows some central hypointensity on T2-w imaging (**B**) with heterogeneous enhancement after administration of contrast (**C**). There is diffusion restriction of the tumor periphery (**D**, **E**). The coronal 3D MPR image after contrast administration (**F**) shows the asymmetrical thickening and enhancement of the facial nerve due to perineural tumor spread on the left (larger arrowhead) compared to the normal diameter of the right facial nerve (smaller arrowhead). Note also the enhancing tissue around the stylomastoid fossa (arrow)

**Fig. 11:**
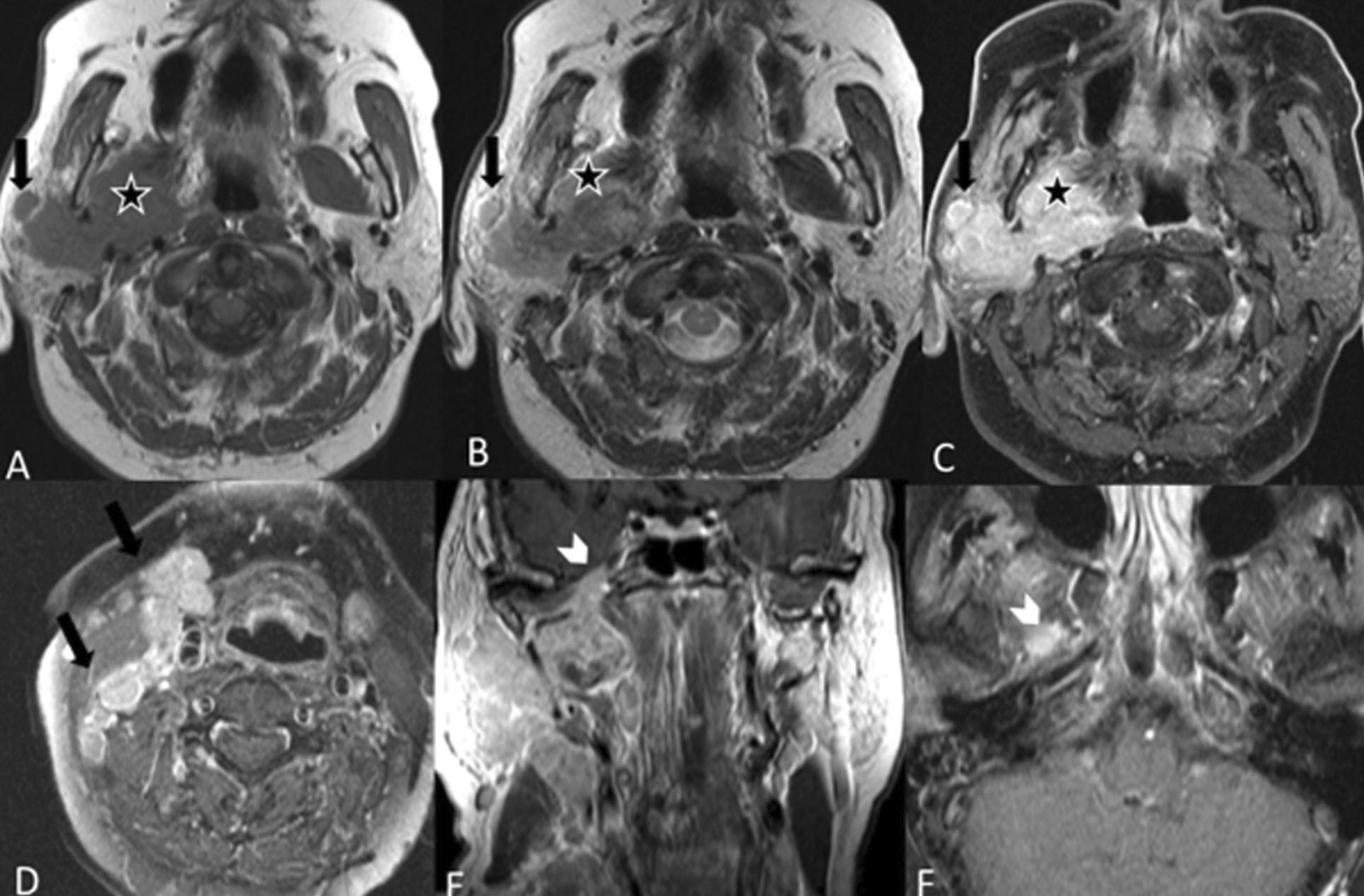
Poorly differentiated carcinoma. A 66-year-old female with swelling of the parotid gland on the right. The axial T1-w image (**A**) shows a large hypointense lesion predominantly in the deep lobe of the parotid gland on the right with extension into the superficial lobe. Note the irregular borders and the small lymph node adjacent (arrow) to the parotid gland. The lesion is slightly heterogeneous on T2-w (**B**) with hypointense areas (below the star). The T1-w fat saturated image after contrast shows strong and diffuse enhancement (**C**). There are many lymph nodes on the right side as seen in the T1-w fat saturated image at a lower level (**D**). There is perineural spread along the mandibular nerve with asymmetrical thickening and enhancement of the nerve in the foramen ovale on the right (arrowhead) as seen in the T1-w fat saturated images after contrast administration in the coronal plane (**E**) and axial plane (**F**)

Malignant tumors include primary parotid malignancies, which are a very heterogeneous group of carcinomas (World Health Organization classification from 2017 lists more than 20 entities of salivary carcinomas). Here we present images of mucoepidermoid carcinoma, adenoid cystic carcinoma, acinic cell carcinoma and parotid carcinoma (Figs. 7, 8, 9, 10, 11). Metastases can also be difficult to differentiate from primary parotid malignancies. It is often not possible to determine the specific histology of parotid tumors solely on the basis of their MRI appearances, but certain findings are indicative of malignancy. An aggressive parotid tumor typically shows low signal intensity on T2-w images and an ill-defined tumor margin with an infiltrative border after contrast administration [22]. Irregular margins and invasion into the adjacent structures can indicate the presence of an infiltrating malignant parotid tumor. The clinical sign that a parotid neoplasm is malignant is facial nerve palsy.

### Mucoepidermoid carcinoma

Mucoepidermoid carcinomas (MEC) are the most common malignant parotid tumors in both children and adults [5, 23]. They most frequently arise in the parotid gland (about 50%), and present as a painless swelling, with or without facial nerve involvement. They often have a cystic component due to their mucin content.

MECs are believed to arise from the pluripotent reserve cells of the excretory ducts and they comprise three different cell types, mucinous, intermediate and epidermoid. Grading reportedly corresponds with the clinical behavior of the tumor. Previous irradiation in the area is a risk factor.

The imaging features depend on the histological type. On MRI, low-grade tumors are well circumscribed with a signal intensity similar to that of pleomorphic adenomas. High-grade tumors, on the other hand, have infiltrating margins with heterogeneous low to intermediate signal intensities on both T1-w and T2-w images (Fig. 7). MECs may show homogeneous or heterogeneous enhancement after contrast administration. Low-grade MECs usually have hyperintense areas on T2-w images, reflecting a cystic architectural pattern due to existence of mucous producing cells. Although some literature describes ill-defined margins as a predictor of high-grade malignancy they can also reflect peritumoral inflammatory change [24].

### Adenoid cystic carcinoma

Adenoid cystic carcinomas (ACCs) are the second most common malignancy of the parotid gland and account for 2–6% of all parotid gland tumors. These tumors arise more often in the minor salivary glands (about 55%) than in the parotid glands. They have the greatest propensity for perineural spread (via cranial nerve VII) and local invasion [25]. Therefore, it is essential to image the facial nerves to assess for perineural spread along the course of the nerve into the mastoid segment of the temporal bone.

On MRI, ACCs are hypointense to isointense on T1-w images and slightly hyperintense on T2-w images with strong enhancement after contrast medium administration (Fig. 8).

### Acinic cell carcinoma

Acinic cell carcinomas of the salivary glands are rare malignant tumors and account for only 2–5% of the primary parotid gland malignancies [26]. They are located almost exclusively in the parotid gland (81–98%) [27] and commonly arise in the parotid tail. They can be bilateral and multifocal. Acinic cell carcinomas are usually painless and slowly growing masses.

On MRI, acinic cell carcinomas usually have non-specific imaging features (Fig. 9), similar to benign tumors such as pleomorphic adenoma or to low‑grade malignant tumors. Therefore, tissue sampling is often required for pathological assessment to establish an accurate diagnosis.

### High-grade carcinoma and poorly differentiated carcinoma

Signs suggesting high-grade malignancies include low to intermediate signal intensity on T2-w, which reflects high cellularity, ill-defined margins reflecting the invasive growth of tumor cells, and frequent nodal involvement reflecting the propensity for lymphatic involvement [22]. Perineural spread is also well seen on MRI and radiological findings include obliteration of fat planes at foramina openings, thickening and increased enhancement of the nerve, and enhancement and muscle changes (Figs. 10, 11).

### Carcinoma ex pleomorphic adenoma

Carcinoma ex pleomorphic adenoma arises from a preexisting pleomorphic adenoma or recurrent benign pleomorphic adenoma. The typical clinical presentation in patients (usually in their 6th–8th decade) with carcinoma ex pleomorphic adenoma is a longstanding history (typically 10–15 years) of pleomorphic adenoma and a sudden period of rapid growth (average, 3–6 months) [28].

On MRI, new aggressive infiltrative margins and rapid enlargement of a preexisting pleomorphic adenoma are features highly suggestive of malignant transformation. Carcinoma ex pleomorphic adenomas are generally heterogeneous on T1-w images due to hemorrhage, necrosis and calcification and are also generally hypointense on T2-w images [28]. They tend to have low ADC values compared to pleomorphic adenomas, which typically have higher values.

### Lymphoma

Primary lymphoma in a salivary gland is rare and accounts for 4.7% of lymphomas at all sites [29]. Non-Hodgkin lymphoma (NHL) presenting in the salivary glands is the most commonly seen lymphoma type and arises within the parotid gland in 75% of cases. The most common subtypes of NHL are extranodal marginal zone B-cell lymphoma of the mucosa-associated lymphoid tissue (MALT) type, follicular B-cell lymphoma and diffuse large B-cell lymphoma. The MALT subtype is more common in a parotid gland. Sjögren syndrome is considered a risk factor [30].

**Fig. 12:**
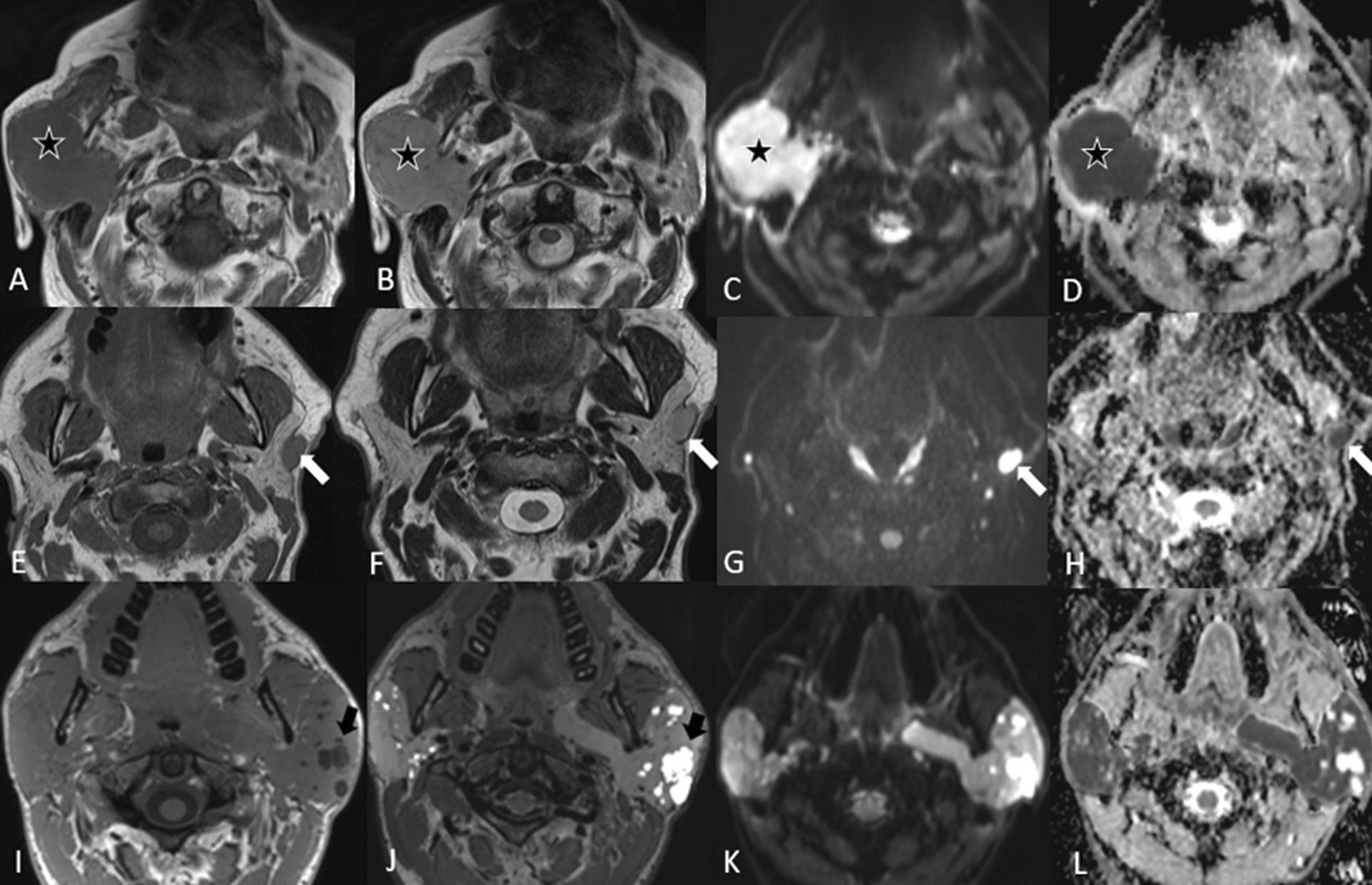
Various radiological appearances of lymphoma: three cases. Patient 1 (**A**–**D**) is an 88-year-old male presenting with a swelling in the right parotid gland, which increased in size for 6 weeks. T1-w axial image (**A**) shows a large, relatively hypointense, homogeneous mass lesion (star) on the right; the lesion is slightly more hyperintense on T2-w (**B**). The lesion shows very high signal on the DWI (**C**) with very low signal on the ADC maps (**D**) revealing the marked diffusion restriction. This was proven by biopsy to be a NHL. Patient 2 (**E**–**H**) is a 66-year-old male with an unclear swelling in the left parotid gland. There were two nodules in the parotid gland on the left. The T1-w image (**E**) shows one lesion (arrow) which is hypointense to the parotid gland and isointense to the skeletal muscles on T1-w, the lesion appears more hyperintense on T2-w images (**F**) but it is still slightly hypointense to the parotid gland. Furthermore, the diffusion-weighted image (**G**) and ADC map (**H**) show marked diffusion restriction. This was a case of follicular NHL. Patient 3 (**I**–**L**) is a 36-year-old male with Sjögren’s syndrome. Axial T1-w (**I**) and axial T2-w (**J**) images show the diffuse enlargement of the parotid glands with multiple small cysts (black arrow). The DWI (**K**) shows some hyperintensity in the parotid gland with a low signal in the ADC map (**L**) indicating diffusion restriction. This was proven to be extranodal marginal zone lymphoma of the parotid gland

Lymphomas generally have low signal intensity on T1-w images and low to high signal intensity on T2-w images, with variable enhancement following contrast administration [31]. Because lymphomas are highly cellular tumors, water diffusion is restricted, making them appear hyperintense on diffusion-weighted imaging (DWI) and hypointense on ADC maps (Fig. 12).

### Metastasis

Metastases of other primary tumors in the salivary glands are relatively rare, but are most commonly seen in the parotid gland. Metastatic involvement of the parotid gland is typically due to cutaneous squamous cell carcinoma and melanoma [32, 33]. The major mechanism of metastasis is via lymphatic spread to intraparotid lymph nodes due to the intense drainage through lymph vessels and the presence of many lymph nodes in the parotid gland. Thus, identification of abnormal‑appearing intraparotid lymph nodes using imaging techniques has important therapeutic and prognostic significance in patients with such cancers.

On MRI, presentation of metastases to intraparotid lymph nodes is similar to metastatic lymph nodes elsewhere in the head and neck area. Progressive enlargement, central necrosis, and ill‑defined margins are all imaging features suggestive of lymphatic metastasis [8]. When large, these look like malignant primary tumors of the parotid gland.

## Advances in MRI technologies: perfusion MRI and radiomics

Dynamic contrast-enhanced (DCE)-MRI provides biomarkers of tissue perfusion with proven utility in oncologic imaging and has been shown to improve distinction of parotid gland tumors [34, 35]. Dynamic contrast-enhanced perfusion MRI, also known as permeability imaging, measures the T1 signal before, during, and after contrast administration and a time–intensity curve is generated from which a transfer constant can be calculated [36]. Four distinct time–intensity curve patterns have been described based on wash-in, wash-out, and time to peak contrast enhancement. Pleomorphic adenomas usually show gradual enhancement. Both malignant tumors and Warthin’s tumors show mostly rapid enhancement, but Warthin’s tumors show a higher wash-out ratio than do malignant tumors [37].

Radiomics is the process of extracting quantitative data from medical images using data characterization algorithms [38]. This is an innovative field in oncological imaging and has also been applied to parotid lesions [39]. The authors performed a retrospective evaluation of the T2 images of parotid lesions from 75 patients. After the regions of interest had been manually drawn, texture analysis was performed with the QUIBIM Precision Platform (QUIBIM SL, Valencia, Spain), which automatically generated 29 quantitative radiomic features including a gray-level histogram and a co-occurrence matrix analysis. The quantitative data were displayed visually using a boxplot, and the unpaired two-sample Wilcoxon rank-sum test was used to find the MRI textural features that could help in the differentiation of benign from malignant tumors, pleomorphic adenoma from Warthin tumor and Warthin tumors from malignant tumors. Receiver operating characteristics curve analyses were done to find the threshold values for the most discriminative features. The results showed that radiomic features based on a histogram and gray-level co-occurrence matrix helped to differentiate between benign and malignant parotid gland tumors, pleomorphic adenomas and malignant parotid tumors and between pleomorphic adenomas and Warthin tumors using the T2-w images. Warthin tumors, however, could not be differentiated from malignant tumors using these parameters [39].

## Conclusions

Certain conventional MRI features can suggest whether a mass is more likely to be a benign or low-grade malignancy or a high-grade malignancy, and adding DWI or advanced MRI techniques like perfusion can aid in this distinction. MRI morphological features such as low signal on T2-w, infiltrative changes or ill-defined margins, changes over time and diffusion restriction can point to the malignant nature of the lesion.

MRI is useful for detection of a lesion or lesions, the localization of the lesion and other associated findings like perineural spread of tumor, lymph node involvement and infiltrative changes of the surrounding tissues. Advanced technologies like dynamic contrat-enhanced perfusion MRI and radiomics show promising results in differentiation of certain pathologies.

However, there are still many lesions that require a fine needle puncture or biopsy for confirmation of the diagnosis. We hope that the examples presented in this pictorial essay will contribute to making the evaluation of these lesions easier.

## Data Availability

The datasets used and analyzed during the current study available from the corresponding author on reasonable request.
